# Multidisciplinary Specialty Teams: A Self-Management Program for Patients With Advanced Cancer

**Published:** 2015-09-01

**Authors:** Christine Tocchi, Ruth McCorkle, M. Tish Knobf

**Affiliations:** Duke University School of Nursing, Durham, North Carolina; Yale University, West Haven, Connecticut

## Abstract

Self-management has been shown to be an effective intervention to enable and empower patients with chronic illness to manage their health. Taking Early Action to Manage Self (TEAMS) is such an intervention, providing education and support to patients with advanced solid tumors to develop self-management skills. We conducted a study and surveyed health-care providers about their perceptions of multidisciplinary teams on the outcomes of this TEAMS intervention as well as factors that may influence its adoption into practice. The majority of respondents reported that the TEAMS program was feasible to practice and well suited to their patient population. In this article, the full results of this survey are presented, along with the emerging themes of empowerment and improved communication between patients and providers. In addition, facilitators and barriers to its adoption are explored. Although providers supported the adoption of the TEAMS program, provider resources to implement and maintain it need to be addressed prior to its widespread adoption.

Self-management has been used as a model of care to enable and empower patients to manage their health (Lorig & Holman, 2003). Self-management involves the formation of partnerships among providers, patients, and families to achieve patients’ own health goals, including the management of symptoms associated with cancer care. In a systematic review of the self-management literature, interventions in cancer care have resulted in less symptom distress, better problem-solving skills, and improved communication among patients, caregivers, and providers (McCorkle et al., 2011). With the guidance of advanced practice nurses (APNs), patients who have been taught symptom and self-management have reported less symptom distress associated with treatment, improved quality of life and mood, and longer survival (McCorkle et al., 1989; Temel et al., 2010).

To meet the physical and psychological needs of patients with advanced cancer, McCorkle and colleagues (2014zzzNo2014) evaluated an extensively tested nursing intervention with postsurgical cancer patients newly diagnosed with advanced solid tumors. The Taking Early Action to Manage Self (TEAMS) intervention provided education and support to late-staged patients to develop self-management skills.

A complete description of the study is published elsewhere (Ercolano, Bai, Lazenby, Ma, & McCorkle, 2013). Briefly, the study was a randomized clinical trial utilizing cluster randomization of ambulatory oncology clinics at a large metropolitan cancer center. The intervention patients received care from the multidisciplinary team coordinated by an APN. The intervention comprised five clinic visits and five telephone calls over 10 weeks. The APNs assessed patients’ current and anticipated needs, provided direct service, made additional referrals to community agencies as necessary, and provided educational strategies to convey factual information about disease and treatment effects and problem-solving techniques to enhance self-management. Care was coordinated with the total multidisciplinary team to meet patients’ needs. The attention control group received usual care provided by the multidisciplinary team in the specialty clinics and may or may not have been evaluated by an APN. Both groups were taught how to use the Symptom Management Toolkit (SMT), a resource manual that describes and offers evidence-based self-management strategies for 28 common symptoms and problems associated with cancer treatment (Given, Given, & Espinosa, 2003).

Study results revealed no significant differences between groups at baseline and at 3 months on six patient outcomes: emotional distress, symptom distress, self-reported health, quality of life, uncertainty, and self-efficacy. However, both groups reported significant improvements on two of the outcomes: health distress and function (McCorkle et al., 2015).

In addition to evaluating the results of the self-management intervention on health outcomes, a specific aim of the TEAMS study was to explore the multidisciplinary team’s perception on components of the intervention that influenced health outcomes as well as facilitators and barriers to its adoption into clinical practice. Inclusion of research and clinical team members in the discussion of study results fosters dissemination, which enables groups to become aware of, obtain, and make use of information (Freemantle & Watt, 1994). The promotion of quality, innovative practice requires team members to be aware of research, able to access information on research findings, and able to interpret information.

Effective dissemination can support multidisciplinary teams to share information about new health-care developments and enable health systems to make decisions regarding effectiveness and cost-effectiveness of health-care interventions (Scott & McSherry, 2009). With finite health-care resources, it is imperative for researchers to examine the perceptions of health-care teams on the effects of research interventions and the feasibility of adopting interventions into practice. The purpose of this article is to explore the perceptions of the members of the multidisciplinary team on the care they provided to study participants and factors that may influence adoption of the program into practice.

## Design and Methods

Quantitative and qualitative data were collected to assess providers’ perceptions of the TEAMS intervention and explore the adoption of the study intervention to practice. A panel of five research members utilized an iterative process in the development of the questions. The final product was a 15-item, semistructured questionnaire representing four areas of adoption: awareness of the TEAMS study, evaluation of the intervention, utilization of the intervention, and description of the health-care provider’s role within the health-care institution. Each question was answered with yes, no, do not know or were open-ended to allow for participant input and elaboration of responses.

Between August 2012 and August 2013, 24 health-care providers who cared for patients in both intervention and enhanced control groups were interviewed (11 intervention; 10 control; 3 both intervention and control). The interviews were completed by one of the investigators (CT) and scheduled to begin at the completion of the intervention with the last patient enrolled. The interviews were audiotaped, listened to in their entirety, transcribed verbatim, and relistened to in order to compare the tape-recorded interview with the transcribed document. Any discrepancies found between written and taped responses were corrected.

Descriptive statistics were used to analyze all multiple-choice answers. Overall frequencies for each answer and means by discipline were conducted. Content analysis was utilized to describe provider responses. Two investigators (CT, TK) reviewed each transcript as well as coded and compared the codes of the first five transcribed interviews. A code key was then created inductively from the initial five interviews. As coding of additional transcripts proceeded, new data were constantly compared with previous codes. When all transcripts were completed, a final key code was reapplied to each transcript. Any discrepancies among investigators were resolved by careful review, negotiation, and consensus.

## Results

The sample consisted of 24 oncology health-care providers in nursing, care management, social work, and medicine (see Table 1). Providers included registered nurses (n = 6, 24%), APNs (n = 6, 24%), physician assistant (n = 1, 4%), care managers (n = 2, 8%), social workers (n = 4, 16%), and oncology physicians (n = 5, 21%). All providers worked in a large academic cancer center within collaborative multidisciplinary specialty teams to provide patient-centered care. The specific multidisciplinary cancer clinics in the study included gynecologic, lung, head/neck, and gastrointestinal.

**Table 1:**
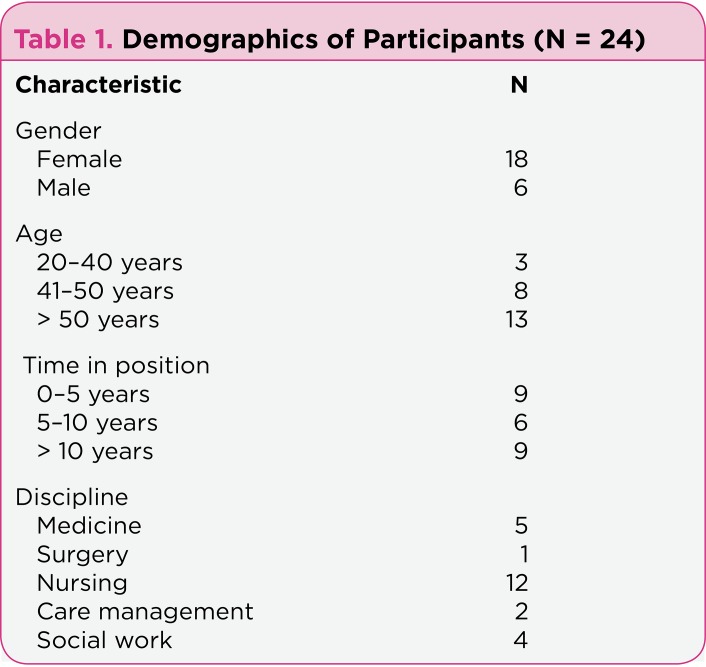
Demographics of Participants (N = 24)

The majority of the participants (n = 23, 96%) knew of the TEAMS study regardless of whether their patients were in the intervention or the enhanced control groups, and about half noted the difference between intervention and control, specifically better perceived health-care utilization among the intervention participants. The participants reported that the TEAMS program was feasible to integrate into practice (n = 15, 63%), judged the intervention to be well suited for their patient population (n = 20, 83%), and stated they practiced in a team model (n = 21, 88%). Data suggested that one-third of the providers perceived that the intervention group had better communication and care coordination than the enhanced control group (see Table 2).

**Table 2:**
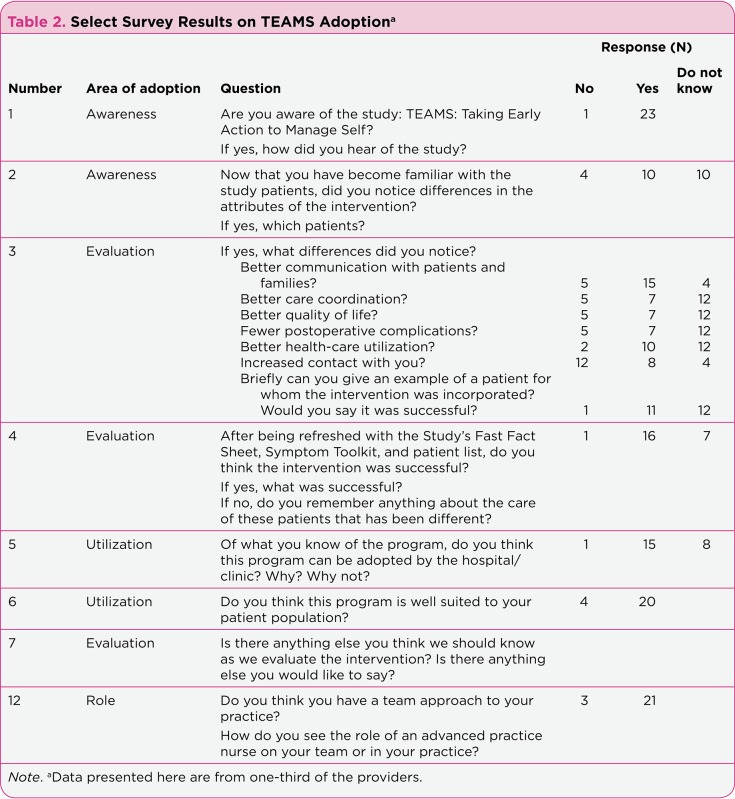
Select Survey Results on TEAMS Adoption

## Outcomes Assessment and Intervention Adoption Into Clinical Practice

Analysis of the qualitative data from the interviews resulted in three themes related to the perception of providers on patient outcomes and the patient/provider relationship: empowerment, communication, and evidence-based practice. Provider participants were also asked about adoption of the study intervention into clinical practice and to identify potential facilitating factors and barriers to its adoption.

**Empowerment**

Many providers (n = 14, 58%) identified that the TEAMS intervention provided by the APNs empowered patients. The support of an APN, the monitoring of patients’ status, and information within the SMT encouraged patients to actively participate in their care. Increased strength, self-confidence, growth, gaining a voice, and ownership of care characterized the providers’ perception of patient empowerment. They also reported that patients showed less anxiety, improved skills to effectively navigate the health system, and improved quality of life. These perceptions were consistent with patients’ self-reports on the clinical outcomes.

**Communication**

Over half of the providers (n = 15, 62.5%) stated that the intervention improved communication. As the SMT was a critical component of the study, providers reported that it was effective for initiating conversation on symptoms and self-management of symptoms. One provider said, "There was an increase in patient’s ability to know when to call. The (patients) would call with a specific question, and they knew when to call for additional treatment options."

Providers also expressed that the SMT supported the use of consistent symptom terminology among providers, patients, and family members. Another provider said, "There was an increased awareness of patients about how to communicate (symptoms) with caregiver and family."

However, a minority of providers (n = 5, 21%) did not observe an improvement in communication in the intervention group. These providers, from medicine and care management, stated that they already had good communication with their patients, and the SMT did not enhance their ability to talk. Two of these providers were from social work and thought that the SMT was redundant with information that patients already received.

**Evidence-Based Practice**

Over half of the providers (n = 16, 66%) supported the incorporation of the SMT for use in their practice because it provided patients and families with evidence-based strategies to manage symptoms. They recognized that the SMT helped to palliate patients’ symptoms. Incorporation of palliative symptom management early in cancer care provided patients with evidence-based knowledge they could use to self-manage their symptoms.

Two comments from providers follow: "The SMT provided a good resource of evidence-based treatment and prevents patients from resorting to the Internet for information." "It is an organized program. It is inclusive and (it is) good to have (evidence-based treatments) in book fashion for ease of use (i.e., for both patients and caregivers)."

**Facilitators and Barriers to Adoption of TEAMS in Clinical Practice**

The health-care providers supported the empowerment and improved communication of the study participants as a result of the intervention, which led to more effective self-management. The program was described as a well-designed intervention with the potential to improve patient outcomes and decrease health-care utilization and costs. One provider stated, "Research supports the importance of self-management to improve health outcomes, and this program would do this."

Also, the providers expressed several concerns for the adoption of TEAMS into clinical practice (see Table 3). They mentioned the resources required for its initiation and maintenance as a barrier. One provider commented, "It [the TEAMS program] is time-consuming to enact and limits our time with other patients. Patients with advanced cancer need additional time for us to address their needs." Another provider added, "It [the TEAMS program] requires the hiring of additional personnel that the hospital will not agree to, but referral to the palliative care team is an alternative."

**Table 3:**
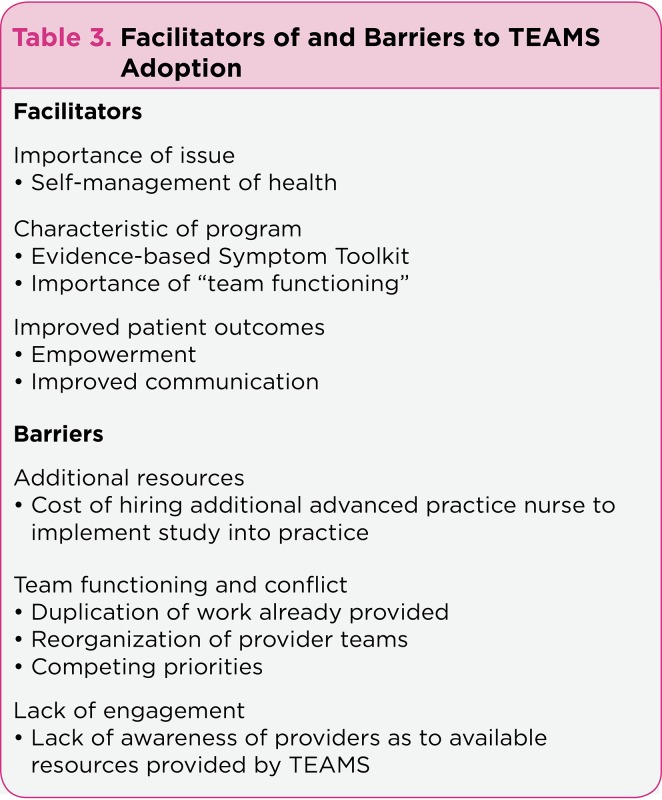
Facilitators of and Barriers to TEAMS Adoption

Another hindrance to the intervention’s implementation seemed to center on competing priorities and the need for reorganization of team members. One provider elaborated on this thought: "We often need to address so many medical problems that we do not have the time to educate or counsel our patients as we would like, and patients with advanced cancer take longer. We also need to spend time with family members, time we just don’t have."

Another barrier identified by providers focused on role conflict. Three providers reported that symptom management and communication were within the scope of practice and responsibilities of social work, and that the adoption of TEAMS would duplicate efforts. One provider added, "This model of care is the domain of social work and is already repeated four to five times."

## Discussion

The objective of this study was to explore the perceptions of health-care providers on outcomes of a self-management intervention for patients with advanced cancer and the factors that may influence adoption of the program to practice. The majority of providers interviewed reported that the TEAMS study was innovative and demonstrated scientific rigor. The incorporation of SMT by APNs provided an innovative approach to self-management in patients with advanced cancer. The multidisciplinary team noted the TEAMS intervention: (1) empowered patients to participate in their health care; (2) enhanced communication between patients and providers; (3) provided patients evidence-based management; and (4) led to self-management of symptoms associated with advanced cancer.

The clinic team providers made clear the importance of TEAMS to empower patients with advanced cancer. They recognized that patients diagnosed with late-stage cancers and comorbidities may be at greater risk of developing treatment related-problems and complications compared with patients with early-stage cancers. The support and education provided by APNs enabled patients in this study to develop confidence (power) within themselves and to use this power to gain mastery in the management of their health. Hibbard, Mahoney, Stock, and Tusler (2007) reported interventions that encourage patients to be involved in their own care enable (empower) patients.

In our study, the end result of empowerment was reported as self-management of symptoms and improved quality of life. Self-management of health has demonstrated the ability to enable patients to develop the skills and coping strategies needed to manage their symptoms. If patients’ symptoms are managed, they are more likely to be able to care for themselves and remain independent and functional. Self-management entails interactive learning, problem-solving, decision-making, action-planning, and using a support system for change. These self-management skills were incorporated into the TEAMS study and accomplished through the clinic APNs’ ability to support the multidisciplinary staff to educate and support the patients (McCorkle et al., 2011).

In the 1980s, McCorkle et al. (1989) documented that early palliative care had a positive impact on forestalling symptom distress in patients diagnosed with progressive lung cancer. Approximately 20 years later, Temel and her team (2010) found similar results. Early palliative care led to significant improvements in both quality of life and mood.

The role of nurses in the development of self-management skills in patients with cancer has been well established in other studies (Braden, Mishel, & Longman, 1998; Badger, Braden, & Mishel, 2001; McCorkle, Tang, Greenwald, Holcombe, & Lavery, 2006; Miaskowski, Dodd, & Lee, 2004; Sikorskii et al., 2007).

For instance, Given and colleagues (2004) developed a nurse-delivered intervention to reduce the severity of physical and psychological symptoms for patients receiving chemotherapy. The nurses provided standardized information consisting of symptom management, counseling, and problem-solving approaches. Results indicated decreased symptom severity for patients in the intervention group (*p* = .01). That intervention was further developed to include the SMT to support the management of common symptoms associated with chemotherapy (Given, Given, Sikorskii, & Hadar, 2007) and resulted in a clinically significant reduction in symptom severity (*p* = .01).

Taking early action to manage symptoms associated with cancer and cancer treatment has been shown to improve quality of life, reduce complications, and result in fewer hospitalizations (Sikorskii et al., 2007). Patient-provider communication is an integral part of symptom management. Patients who can articulate the distresses associated with their symptoms are more likely to acknowledge health problems, discuss health concerns with providers, understand their treatment options, modify their behavior accordingly, and follow their medication schedules. In fact, research has shown that effective patient-provider communication can improve a patient’s health quantifiably (Ong, de Haes, Hoos, & Lammes, 1995; Stewart, 1995; Makoul & Curry, 2007).

Good clear communication produces a therapeutic effect that has been validated in controlled studies. The use of the SMT provided a means for patients and providers to initiate conversations about their symptoms utilizing a common vernacular with specific terminology (Given et al., 2007). These conversations often facilitated discussions about other problems. In the current study, both education and support provided by APNs in addition to the SMT resulted in more effective communication with health-care providers (McCorkle et al., 2014zzzNo2014).

One advantage of the TEAMS intervention was the use of evidence to support self-management. Evidence-based practice is the gold standard for health care to support quality patient care. Evidence-based care involves the collection, critical analysis, and integration of valid and applicable research into patient care. Unfortunately, patients may obtain misleading and erroneous information from a variety of sources. For example, the Internet is often a source of health information for patients, and they may not have the knowledge or skills to interpret what is presented and to differentiate its applicability to their situation. In this study, regular contact with APNs to discuss the evidence and management of options contributed to higher-quality patient-centered care and high satisfaction.

The majority of providers supported the adoption of TEAMS. Self-management associated with advanced cancer and its treatment was deemed an essential aspect of care by providers.

However, the cost, role conflict, and lack of engagement were identified as barriers to integrating the intervention into routine daily clinical practice. Cost was a reported concern related to time and a perceived need to hire additional APNs to implement and adopt the program. The barriers of time and cost were expected findings, since both are important factors in the adoption of any health-care program. Although cost can be a significant barrier, it must be considered as net cost relative to patient outcomes.

Self-management education has been determined as essential and a standard of practice known to effect positive outcomes (Knobf et al., 2012). Given and colleagues (2009) demonstrated that patients receiving five contacts from a nurse via telephone intervention at 16 weeks had a reduction in symptom severity and an adjusted mean of 1.1 days in the hospital compared with the control group, which had a mean of 2.23 hospital days. Reductions in hospitalization related to reduced symptom severity suggest that the telephone intervention may produce a net savings over the cost of its development and implementation. Evidence of improved patient health outcomes and patient satisfaction are factors administrators consider in the adoption of care programs, as hospitals strive to maintain high patient approval and innovative programs to improve patient care (Donovan & Knobf, 2009).

Providers discussed role conflict as a barrier to implementation of research findings into practice. The negative effect of role conflict on the function of multidisciplinary teams and patient outcomes has been noted in the literature.

Jones (2006) proposed the need for full participation and role clarity of all team members for multidisciplinary teams to function effectively. Similarly, Mitchell and colleagues (2012) stressed the need for full participation of all team members as necessary for multidisciplinary teams to function effectively, and status differences among team members may depress team functioning.

Possible explanations for role conflict include ambiguity of each team member’s function; lack of communication among team members; and discipline boundaries. Inherent to health-care teams is reduction in autonomy with an emphasis on collaboration. There will most likely be a certain overlapping of roles. This however, can be a positive function of multidisciplinary teams if team members view the overlap as a desirable component to quality health care. Role overlap may support improved patient communication through repetition of health information, be used as a mechanism for competency, and encourage trusting interdisciplinary relationships.

Lack of engagement was the third barrier to adoption identified by the providers. Although only a minority of providers stated they were not actively engaged in the study, they voiced concern over the lack of awareness of available resources provided by the TEAMS intervention. It must be noted that those providers who lacked awareness or engagement in the study were in the control group and would not have been privy to all study components. However, for adoption of TEAMS, it will be necessary to address the intervention protocol, results, and available resources at the provider and organizational levels to effectively disseminate research findings. The barriers identified by the multidisciplinary team are not unique to self-management advanced cancer care.

Inclusion of the perceptions of the team members was a specific aim of the TEAMS study to explore effectiveness, dissemination, and adoption of the study intervention. Researchers often do not have the opportunity to reenter the study setting to share study findings and explore clinical team members’ perceptions on the health outcomes of an intervention and opportunities for dissemination and implementation. Promoting dialogue on the intervention process and outcomes encourages health-care providers to critique the effectiveness of the intervention and the appropriateness of the intervention for their patient population and organization (Bradley, McSherry, & McSherry, 2010). Clinical team members are often aware of the constraints of implementing innovative intervention programs and can offer specific concerns related to their institution and patient population. The semistructured interviews of this study allowed the researchers to gather information on the aspects of the intervention that is most beneficial to patients as well as components that might foster or impede its adoption (Glasgow, McKay, Piette, & Reynolds, 2001).

Inclusion of clinical team members in post-study discussion has been shown to promote positive attitudes and support for adoption of intervention programs and for clinical providers to become adoption champions within an organization (Carlfjord, Lindberg, Bendtsen, Nilsen, & Andersson, 2010; Huij et al., 2013). The perceptions of the multidisciplinary specialty team in this study will be crucial to the dissemination of the TEAMS intervention. The combination of study findings and support from multidisciplinary team members will allow researchers to present comprehensive information to health-system administrators to influence adoption of effective innovative programs.

**Study Strengths and Limitations**

The strengths of the analysis of the TEAMS study were the consistent APN contact to support self-management and early palliative care to patients with advanced cancer and the incorporation of an evidence-based SMT into their care. To our knowledge, this may be the only study to explore the perceptions of oncology multidisciplinary team members on the health outcomes related to the study and facilitators and barriers to intervention adoption.

A limitation of the TEAMS study was the potential contamination across clinics due to changes in staffing over the 3 years of the study. Specifically, APNs worked in clinics that included both intervention and enhanced control groups and covered for each other during holidays and vacations. Despite this concern, these providers stated they could differentiate outcomes based on their care for specific study patients.

The information collected was based on reflections by the providers, which may have caused recall bias or failure to disclose all information. There is also the possibility of a positive bias by providers who supported the use of APNs to promote self-management in patients with advanced cancer. However, the analysis identified both positive and negative intervention adoption perspectives to balance possible bias.

## Conclusion

Patients are living longer with cancer, and cancer is being viewed as a chronic health condition. Therefore, it is important to identify the needs of patients living with advanced cancer, develop effective interventions, promote self-management, and adopt evidence-based management into clinical practice. The TEAMS study demonstrated the ability to empower patients to actively participate in their health, promote open communication between providers and patients, and contribute to evidence-based care for patients with advanced cancer. Team providers supported the adoption of TEAMS, but provider resources to implement and adopt the intervention need to be addressed for its more widespread translation into practice.

**Acknowledgment**

Research reported in this publication was supported by NIH RO1NR011872 McCorkle.
